# Associations of multidimensional health literacy with reported oral health promoting behaviour among Slovak adults: a cross-sectional study

**DOI:** 10.1186/s12903-018-0506-6

**Published:** 2018-03-14

**Authors:** Eva Cepova, Martina Cicvakova, Peter Kolarcik, Neda Markovska, Andrea Madarasova Geckova

**Affiliations:** 1 CoHeReNT - Community Health Reasearch Network, Faculty of Medicine, P. J. Safarik University, Kosice, Slovakia; 2 Department of Health Psychology, Faculty of Medicine, P. J. Safarik University, Trieda SNP 1, 040 66 Košice, Slovakia; 3 Olomouc University Society and Health Institute, Palacky University Olomouc, Olomouc, Czech Republic; 4 Department of Stomatology and Maxilofacial Surgery, Faculty of Medicine, P. J. Safarik University, Rastislavova 43, 041 90 Košice, Slovakia; 5 Department of Stomatology, Faculty of Medicine, Slovak Medical University, Limbová 14, 833 03 Bratislava, Slovakia

**Keywords:** Oral health-promoting behaviour, Health literacy, Logistic regression analysis, Adults, Slovakia

## Abstract

**Background:**

Modification of health literacy (HL) is an important factor for improving and maintaining oral health. The aim of the study is to examine the association of HL with oral health-promoting behaviour (OHPB) and assess possible mediating effects of HL on the impact of socioeconomic status on OHPB.

**Methods:**

A cross-sectional questionnaire survey on the Slovak general adult population (*N* = 360, mean age 39) was conducted in 2014 and 2015. The association of HL (9 domains of the Health Literacy Questionnaire) and OHPB was analysed using logistic regression models adjusted for gender, age and educational level. Testing the mediating effect of HL domains between education attainment and OHPB was performed using the Sobel test.

**Results:**

Women and respondents with higher education reported better OHPB. Regular tooth-brushing is associated with better HL in five domains: Feeling understood and supported by healthcare provider, Having sufficient information to manage my health, Activelymanaging my health, Social support for health, Appraisal of health information (Odds ratios (ORs) from 1.64 to 2.33, *p* < 0.05). Using interdental tools is in association with better HL in two domains: Feeling understood and supported by a healthcare provider and Having sufficient information to manage my health (ORs 1.71 to 1.80, *p* < 0.05). Respondents who visited a dentist for prevention score higher in Social support for health (OR 1.79, *p* < 0.05). Using a tongue scraper and single brush and reporting gums bleeding is notstatistically significantly associated with HL. Mediation was confirmed between the effect of respondents’ education on using fluoride toothpaste – mediated respondent’s ability to find good health information. Frequency of tooth-brushing and using interdental hygiene aids were both mediated by patient’s sufficient information to manage health.

**Conclusions:**

Our results indicate HL to be an important factor related to good oral health, and HL should be considered when planning oral health interventions.

**Electronic supplementary material:**

The online version of this article (10.1186/s12903-018-0506-6) contains supplementary material, which is available to authorized users.

## Background

Health literacy has an increasing trend of interest over the last decade. Health literacy is considered as a promising concept and as a tool in health promotion and health improvement in patients with chronic disease and diseases related to patients’ life-style [1, 2]. Although oral diseases can be avoided by proper oral health promoting behaviours and preventive activities, this problem continues to persist in many countries around the world [3].

Health literacy is defined as “the personal, cognitive and social skills which determine the ability of individuals to gain access to, understand, and use information to promote and maintain good health” [4]. Increasing interest in health literacy is driven by evidence showing an association between health literacy and health outcomes. Inadequate health literacy is associated with a wide range of health-related outcomes, including poorer health status, lower use of preventative health care, higher mortality and more hospitalizations [2, 5, 6].

In the field of oral health, narrower concept of health literacy was proposed in literature and utilized in the research [7, 8]. The term of oral health literacy describes the skills and abilities that enable the acquisition and processing of information in association with oral health. A higher level of oral health literacy is important for improving people’s awareness of the presence of oral health complications, tooth cavities, as well as for widening the knowledge about the prevention of these diseases and improve individual’s oral health promoting behaviours [9]. We use Health literacy questionnaire because, based on general and multidimensional health literacy construct [10], instead of the specific oral health literacy tool, because the used scales and questionnaires do not reflect the most recent trends in conceptualisation of health literacy. Tool used for measuring oral health literacy in the research setting are mostly aimed to recognizing words, numeric and reading skills [11], which do not relate to respondents ability to find, comprehend and utilize health related information. This narrowed concept of health literacy presumes two different sets of skills of the individual, one related to oral health and the other related to general health. In general, health literacy consists of skills that affect overall health, including oral health, and using a narrowed concept might be confusing [4]. It is of general interest to focus on general health literacy and its relation to oral health, rather than narrowed oral health literacy. On the other hand, there is noticeable increase of interest in health literacy and oral health in recent years [1, 11]. This is indicated by increasing attention related to health literacy and oral health from academics and also by professionals in dentistry, public health and health care systems [11].

The literature offers only a few studies examining the relationship between health literacy and oral health outcomes [12, 13]. It has been suggested that those with low health literacy are at highest risk for oral diseases and problems [14] and that low health literacy may also be associated with barriers to accessing health care and engaging in oral health promoting behaviours, such as seeking preventive dental care [7]. Oral health is gradually recognized by the professionals as a concept broader than just healthy teeth, because poor oral health has a large impact on an individual’s overall health status [15]. Oral health is understood to be an integral part of general health. Negligence of oral health leads to various health problems and chronic diseases, such as cardiovascular diseases, respiratory diseases and complications during pregnancy (premature delivery and low are born sooner and smaller), which belong among diseases with mass occurrence [16].

Oral health is associated not only with clinical and subjective factors, but also with social factors (socioeconomic status and social capital) and psychological well-being [3].Good oral health enables people to communicate confidently, have an optimal quality of life and maintain positive self-esteem and self-confidence. However, people of adolescent age are already at risk of developing dental caries and periodontal disease because of their high-sugar diets, poor oral hygiene practices and limited use of fluoride tooth paste [17].

Health problems related to oral cavities are persistent not only in Slovakia, but all over the world [18–20]. These problems are alarming and have shown very little improvement during the last decades. According to WHO, 60–90% of school children and nearly 100% of adults worldwide have dental cavities, often leading to pain and discomfort. Severe periodontal disease, which may result in tooth loss, is found in 15–20% of middle-aged (35–44 years) adults. Globally, about 30% of people aged 65–74 have no natural teeth [16]. According to Regional public health authority, a large proportion of the Slovak population (up to 80–90%) suffers from some kind of tooth and periodontal diseases [21].

Our study is the first to use the multidimensional concept of health literacy built into the Health Literacy Questionnaire (HLQ) in association with oral health. The HLQ is a comprehensive measure of HL capable of diagnosing HL needs across individuals and organisations by utilizing perspectives from the general population, patients, practitioners and policy makers [10]. The HLQ covers nine conceptually distinct areas of health literacy to assess the needs and challenges of a wide range of people and organisations. The HLQ is a valid instrument for measuring health literacy in the Slovak Republic, Denmark and Australia [10, 22, 23].

### Aim of the study

The oral health of the Slovak population is alarming and has shown very little improvement during the last decades [24]. Understanding the association of patient’s health literacy and oral health in Slovakia would help with designing and better focusing of future interventions aimed at improving oral health in the Slovak population. The aim of study is to examine the association of health literacy with oral health indicators and further to consider the impact of gender, age and education level on oral health using a population sample of Slovak adults.

## Methods

### Procedure

Data were collected cross-sectionally in private dental clinics and at the 1st Dental Clinic of the UNLP in Košice and Prešov regions in Slovakia from December 2014 to February 2015.We contacted eight dental clinics, and six of them agreed to administer questionnaires to their patients –four in Košice region and two Prešov region. Only 53.5% of the Slovak population participated in a preventive check up in 2013 according to the National Health Information Center [24]. Respondents were approached in waiting room and asked to fill in the questionnaires by research assistants who were available for any questions or problems with answering the questionnaire. Respondents were informed of the purpose of the study and their written informed consent was acquired. Completion of the questionnaire took 15–25 min. The socioeconomic and demographic profile of the respondents corresponds with characteristics of the general population in the eastern part of Slovakia [22].

### Ethical consideration

The ethical approval was obtained on November 29, 2013 from Ethical Committee of the Faculty of Medicine, P.J. Šafarik University.

### Sample

We approached 498 respondents visiting dental outpatient clinics in the given period, 360 of which completed and returned the questionnaire, giving a response rate of 72%. Questionnaires with missing data on age, gender, socioeconomic status, oral health and health literacy were excluded (*n* = 25). The final sample consisted of 335 participants (47.5% males). The age of respondents ranged from 18 to 68, with a mean age of 38 years (SD = 14).

### Measures

The questionnare of items on age, gender, education, oral health-promoting behaviour and health literacy. Respondents were asked about their highest completed education, with four answers offered: (1) primary education, (2) secondary school, (3) primary school without graduation and (4) univesity.Thesewere thendichotomized as (1) no univesity (1 + 2 + 3) and (2) university.

HLQ is a recently developed tool for measuring health literacy in nine domains. These domains enable a detailed profile of the health literacy of individuals, a subpopulation or patients of interest to be derived. The domains of HLQ are as follows: *Feeling understood and supported by healthcare providers (HLQ domain 1), Having sufficient information to manage my health (HLQ domain 2), Actively managing my health (HLQ domain 3), Social support for health (HLQ domain 4), Appraisal of health information (HLQ domain 5), Ability to actively cooperate with healthcare providers (HLQ domain 6), Navigating the healthcare system (HLQ domain 7), Ability to find good health information (HLQ domain 8), Understanding health information well enough to know what to do (HLQ domain 9).* The original HLQ is divided into two parts which differ in response categories. Part 1 (HLQ domains 1–5) has four response catogories rating the extent of agreement (Strongly disagree (1), Disagree (2), Agree (3), Strongly agree (4). Part 2 (HLQ domains 6–9) has five response categories rating the level of difficulty. These five categories originally had the following wording: *Cannot do (1), Very difficult (2), Quite difficult (3), Quite easy (4) and very easy (5)*; this was later changed by the authors to improve the way of responding to items as follows: *Cannot do or always difficult (1), Usually difficult (2), Sometimes difficult (3), Usually easy and (4), Very easy (5)*.

Oral health and oral health-promoting behaviour were measured by five items covering reasons to: a) visit the dentist, b) using toothpaste with fluoride, c) frequency of tooth-brushing, d) gums bleeding, and e) using additional hygiene aids other than a toothbrush and toothpaste in their dental hygiene (see Additional file 1). Respondents were asked about the main reason to visit their dentist. We offered them a list of possible reasons, two of which – preventive check-up and dental hygiene – were considered as preventive behaviour, and six other reasons as non-preventive (making a crown, bridge or dental plate; to fill cavities; endodontic treatment or extraction of a nerve; tooth extraction; other surgical intervention). Respondents were also asked whether they use toothpaste with fluoride (yes/I do not know; I do not use it; I avoid fluoride toothpaste), how frequently they brush their teeth (after each meal; twice a day/once a day; rarely), if their gums bleed while brushing their teeth (never/rarely; sometimes; often). Moreover, a list of other dental hygiene aids – mouthwash, single brush, tongue scraper, interdental floss, interdental thread and electric toothbrush – was provided and they were asked to mark those items they use for dental hygiene. Dental hygiene aids item consisted of two parts– Using interdental floss or thread and Using a scraper or single brush.

### Statistical analysis

As a first step the descriptive statistics for the sample were calculated. Gender and education differences were explored using chi square. As a second step, the association of oral health-promoting behaviour and health literacy was determined using logistic regression. We tested the crude (crude effects are not reported) and adjusted health literacy effect on oral health-promoting behaviour items. Adjustments were made regarding gender, age and education level. Statistical analyses were performed using IBM SPSS 22.0. The testing of the mediating effect of health literacy domains between education attainment and oral health-promoting behaviour was performed using the Sobel test.

## Results

More than half of respondents visited their dentist for a preventive check-up or dental hygiene (see Table 1), reported using toothpaste with fluoride and using interdental floss or thread. Even more respondents, over 70%, clean their teeth at least twice a day (see Table 2). On the other hand, only about one-third of respondents reported no bleeding of gums and the use of a scraper or single brush. Women and university educated respondents reported significantly more frequently sufficient frequency of teeth cleaning (at least twice a day), using interdental floss or thread and visiting a dentist to undergo a preventive check-up or dental hygiene in comparison to men and less educated respondents. Using toothpaste with fluoride was reported by university educated respondents significantly more frequently than by less educated respondents. We also analysed age differences in OHPB. We found only two statistically significant differences between age categories and OHPB: bleeding gums and using interdental floss or thread. Older respondents reported more frequently bleeding gums but also less frequently using of interdental floss. In the rest of surveyed OHPB we did not find statistically significant differences between age groups.

**Table 1:** Frequency of respondents’ reasons for visiting the dentist

	Total		Men		Women	
	*n*	%	*n*	%	*n*	%
Preventivecheckup	188	52.2	70	40.7	118	62.8
Dental hygiene	44	12.2	16	9.3	28	14.9
Toothache	167	46.8	93	54.1	74	39.4
Making a crown, bridge or prosthesis	19	5.3	13	7.6	6	3.2
Makingthefiller	78	21.7	45	26.2	33	17.6
Endodental treatment	10	2.8	6	3.5	4	2.1
Tooth extraction	20	5.6	16	9.3	4	2.1
Other surgical procedure	18	4.2	13	7.6	5	2.7

**Table 2:** Differences in oral health-promoting behaviour by gender and educational attainment (*n* = 335, Slovak adults aged 18 to 68 years, data collected in 2015)

	Dentist preventive check-ups or dental hygiene (yes)		Toothpaste with fluoride (yes)		Frequency of tooth-brushing (at last twice a day)		Bleeding of gums (never)		Using interdental floss or thread (yes)		Using a scraper or single brush (yes)	
	*n*	%	*n*	%	*n*	%	*n*	%	*N*	%	*n*	%
Gender												
Men	78	49.06	83	52.2	115	72.3	43	27.0	67	42.1	38	23.9
Women	123	59.89	101	57.4	147	83.5	51	29.0	110	62.5	37	21.00
Difference significance	***				**				***			
Education												
No university	136	57.6	113	47.9	175	74.2	69	29.2	110	46.6	47	19.9
University	65	65.7	71	71.7	87	87.9	25	25.3	68	67.7	28	28.3
Difference significance	***		***		**				***			
Age categories												
18–30 years	75	55.1	76	55.9	114	83.8	36	26.5	83	61	23	16.9
31–44 years	51	60.7	47	56	64	76.2	26	31	47	56	19	22.6
45–64	67	65.7	56	54.9	75	73.5	24	23.5	42	41.2	28	27.5
≥ 65	6	54.5	4	36.4	7	63.5	7	63.6	3	27.3	5	45.5
Difference significance							*		**			

Chi-square, *p < 0.05, **p < 0.01, ***p < 0.001

After descriptive analyses the associations between HLQ domains and outcomes of oral health were tested. Crude and adjusted HLQ domain effects on the outcomes were analysed (see Table 3).

**Table 3:** The association between health literacy domains and oral health adjusted for gender, age and educational level. Logistic regression. (*n* = 335, Slovak adults aged 18 to 68 years, data collected in 2015)

		Dentist preventive check-ups or dental hygiene (yes)	Toothpaste with fluoride yes	Frequency of tooth-bushing (at least twice a day)	Gums bleeding (never)	Using interdental floss or thread (yes)	Using a scraper or single brush (yes)
Model		OR (95% CI)	OR (95% CI)	OR (95% CI)	OR (95% CI)	OR (95% CI)	OR (95% CI)
HLQ 1	Feel understood and supported by Healthcare provider	1.12 (0.72–1.74)	1.30 (0.84–2.01)	1.90 (1.13–3.18)^**^	0.88 (0.55–1.40)	1.80 (1.14–2.86)**	0.66 (0.40–1.11)
HLQ 2	Have sufficient information to manage health	0.84 (0.56–1.27)	1.16 (0.78–1.74)	1.64 (1.01–2.66)^*^	0.98 (0.63–1.52)	1.71 (1.12–2.61)**	1.01 (0.62–1.63)
HLQ 3	Actively managing my health	1.37 (0.88–2.13)	1.02 (0.66–1.57)	2.33 (1.37–3.96)^***^	1.16 (0.72–1.86)	1.26 (0.80–1.97)	1.12 (0.67–1.88)
HLQ 4	Social support for health	1.79 (1.13–2.84)***	1.18 (0.75–1.84)	1.73 (1.02–2.92)^*^	1.10 (0.68–1.78)	1.09 (0.69–1.73)	1.08 (0.63–1.85)
HLQ 5	Appraisal of health information	0.92 (0.60–1.42)	1.00 (0.65–1.52)	2.18 (1.29–3.70)^***^	1.21 (0.76–1.91)	1.27 (0.82–1.96)	1.01 (0.61–1.66)
HLQ 6	Ability to actively cooperate with health care providers	0.94 (0.68–1.30)	1.35 (0.97–1.86)	1.34 (0.92–1.97)	1.05 (0.75–1.49)	1.15 (0.83–1.59)	0.85 (0.59–1.24)
HLQ 7	Navigating the healthcare system	0.87 (0.63–1.20)	1.35 (0.98–1.86)	1.43 (0.98–2.10)	1.00 (0.70–1.85)	1.05 (0.76–1.45)	0.94 (0.65–1.37)
HLQ 8	Ability to find good health information	0.97 (0.71–1.32)	1.48 (1.08–2.03)**	1.32 (0.92–1.89)	1.23 (0.87–1.73)	1.25 (0.91–1.73)	1.00 (0.70–1.44)
HLQ 9	Understanding health information well enough to know what to do	0.98 (0.70–1.37)	1.38 (0.99–1.93)	1.37 (0.93–2.02)	1.08 (0.76–1.54)	1.29 (0.92–1.81)	0.88 (0.60–1.29)

Model – adjusted effect (for gender, age and education level of particular domain of health literacy on the outcome variable

^*^p < 0.05, ^**^p < 0.01, ^***^p < 0.001, *OR* odds ratio, 95% CI confidence interval

### Prevention

We found a statistically significant association between Social support for health (HLQ domain 4) and visiting the dentist for a preventive check-up or dental hygiene procedure. Respondents with better social support for health were significantly more likely than respondents with a lower score on this domain to attend a preventive check-up or dental hygiene procedure.

### Using fluoride toothpaste

The adjusted model found one statistically significant association in Ability to find good health information (HLQ domain 8), which associated with reported use of fluoride toothpaste.

### Frequency of tooth-brushing

Respondents who reported higher levels of: Feel understood and supported by healthcare provider (HLQ domain 1), Have sufficient information to manage health (HLQ domain 1), Active management of their health (HLQ domain 3), 4. Social support for health (HLQ domain 4) and Appraisal of health information (HLQ domain 5) are statistically significantly more likely to report brushing their teeth at least twice a day in the adjusted logistic model.

### Dental hygiene aids (interdental brush/thread)

We found statistically significant associations of two domains: Feeling understood and supported by healthcare provider (HLQ domain 1) and Have sufficient information to manage health (HLQ domain 2) with the use of interdental hygiene aids. Respondents reporting higher levels in those domains reported using interdental brush or interdental thread as addition to standard tooth-brushing.

### Gums bleeding and using a single brush or tongue scraper

We did not find statistically significant associations between bleeding gums, using single brush/tongue scraper and health literacy domains.

Analysis of the mediating effect of health literacy between education and oral health-promoting behaviours did not show any statistically significant results in most of the associations tested, the exceptions being three in which the mediating effect was statistically significant. Specifically, mediation was confirmed between the effect of a respondent’s education on using fluoride toothpaste – mediated by Ability to find good health information (HLQ domain 8) (Sobel test = 1.95, SE = 0.11, *p* < 0.05), while frequency of tooth-brushing and using interdental hygiene aids (interdental brush/thread) were both mediated by Have sufficient information to manage health (HLQ domain 2) (Sobel test = 2.01, SE = 0.23, *p* < 0.05 and Sobel test = 2.20, SE = 0.21, *p* < 0.05, respectively).

## Discussion

The aim of the presented study was to examine the associations of health literacy and oral health-promoting behaviour of a sample of the Slovak population with regards to gender, age and education level using the Slovak version of the HLQ. We found that women and respondents with higher education to be more likely to visit the dentist for preventive check-up, brush their teeth regularly and use interdental toothbrush and thread. We can conclude that women and university educated respondents tend to have better oral health-promoting behaviour, regardless of age. Higher health literacy is also associated with better oral health-promoting behaviour, but not all HL domains were associated significantly. Higher levels in HL domains: “Feeling understood and supported by healthcare provider”, “Having sufficient information to manage health”, “Active managing of one’s health”, “Social support for health” and “Appraisal of health information” were associated with tooth-brushing at least twice a day.

Our findings about differences between genders and educational levels are consistent with studies that showed oral health promoting behaviours [25]. Female gender and a higher educational level were associated with a better of oral health-promoting behaviours than were male gender and lower educational levels, which is in line with studies concluding that unhealthy behaviours are more common among men and those in the lower education group [26–28].Among most frequently reported risk factors for poor oral health and OHPB appeared factors such as male gender, black race, low education, low economic status and no access to dental care [25, 29].

Low socioeconomic status is related not only with worse oral health but also with lower health literacy [22, 30, 31]. Thus health literacy might mediate the effect of socioeconomic status on the oral health of respondents; however, our analyses do not fully support a mediating effect of health literacy domains between education attainment and oral health-promoting behaviours. We confirmed the association of education attainments (SES indicator) and oral health-promoting behaviours to be mediated by specific HL domain in only three cases.

Maintaining good oral health requires an individual to understand and to act on health information, whether communicated verbally or in written form [31]. The association between health literacy and oral health is supported by our findings, where several specific domains were related to oral health outcomes. Our results indicate that different oral health indicators challenge specific health literacy domains, but such differentiation of the health behaviours has not yet been reported in other studies [12]. It is interesting that three domains dedicated to relatively advanced skills were not significantly related statistically to oral health outcomes (i.e.: Ability to actively cooperate with health care providers, Navigating the healthcare system, understanding health information well enough to know what to do).

Regular visiting the dentist for prevention or dental hygiene is generally accepted as being among the important steps needed to maintain an individual’s oral health. Our study shows that the main reason for visiting a dentist on a sample of Slovak adults is still an oral health problem or emergency treatment. This is similar to the finding of Devraj, who reported extraction of a tooth as the main reason for a dentist visit in more than 40% of the study sample [32]. On the other hand, more positive outcomes were reported by an EU report, which states that most Europeans seem to consult a dentist for preventive reasons and not for emergency treatment: 50% of the people interviewed during the survey said that the last time they visited a dentist was for a check-up, an examination or a cleaning. A third went for routine treatment, and only one in five went for emergency treatment [33]. Slovak adults do not yet follow this European trend.

Another important step in maintaining good oral health is regular tooth-brushing and using toothpaste with fluoride. We found that respondents with higher HL domain Ability to find good health information (HLQ domain 8), reported using toothpaste with fluoride more frequently. It seems that the ability to get reliable health information differentiates respondents who report using fluoride toothpaste and being aware that fluoride in toothpaste is important in preventing tooth decay. Regular tooth-brushing was related with a group of HL skills. It seems that such an oral health promoting activity requires not just sufficient health information, appraisal of health information and active health management, but also a supportive social environment. The study of Cruz (2014) supports the notion that higher levels of HL contribute to regular teeth cleaning and better oral health [34]. We could conclude that higher HL is linked with a more responsible individual’s approach to his or her oral health and oral health-promoting behaviours.

Very few studies have been published on health literacy in a dentistry setting. The limited research that has been done focuses mostly on assessing the ability to read dental educational materials and consent forms [35], while little research has been undertaken to examine health literacy levels in association with oral health. Oral health literacy tools have been used since 2007 [11]. Existing OHL instruments are limited in their objectives, and they measure either the ability of a person to read specific dental health vocabularies or the ability of a person to read and comprehend written oral health information and to calculate the numbers. The most widely used oral health literacy measurement tools are based on either the Rapid Estimate of Adult Literacy in Medicine (REALM) [36] or the Test of Functional Health Literacy in Adults (ToFHLA) [37]. The oldest measuring tool OHL is REALD-99 focused on item recognition [38]. The systematic review showed strengths and weaknesses of measuring tools OHL. Early tools attracted the same criticisms directed at the general health literacy versions, in that they were largely word recognition tools that did not actually measure oral health literacy per se, rather they provided an approximate measure of reading skills relative to oral health content [39]. The authors conclude that adding two new measures (listening and decision-making) improves the performance and quality of the existing instruments. The most recent OHL measuring tool is HELD (2013) is already based on multidimensional nature of oral health literacy [40] but it is still not so broad as conceptualisation of HL which was used in designing HLQ [10]. Furthermore, according to the review of Hongal et al., the health literacy barrier to oral health has been largely invisible until recently because it was seldom recognized and poorly understood by professionals, and many health care providers could not address the health literacy needs of their patients [8].They also tend to use materials that were readily available but difficult to understand, and this made patients reluctant to admit that they did not comprehend the information presented. Many patients were also found to be uncomfortable asking questions or requesting more information from their dentists [8, 29].

Oral diseases pose a serious risk for an individual’s overall health status and also for public health. Numerous interventions focus on improvement of oral health and promoting preventive actions, but with limited success. It might be beneficial to include patients’ health literacy as a key factor in designing future interventions. Our study may inspire further research and interventions to utilize health literacy assessment, which enables individuals with limited health literacy to be identified and provides information about the strengths and weaknesses of current oral health-promoting programs and interventions. Our study provides information not just on patients’ weaknesses in oral health-promoting behaviours but also data about respondents’ health literacy levels and their associations with oral health outcomes. Information about patient’s health literacy may be utilized in preparing more efficient interventions aiming in change of oral health promoting behaviours and thus improving population oral health. There are various approaches based on health literacy concept. One of them, which we consider as an example of a very promising intervention based on health literacy assessment, is the Ophelia Approach, which might help to advance the understanding of health literacy and shows how it can be used to improve health outcomes [41]. It involves the collaboration of a wide range of community members, community leaders, and workers to develop health literacy interventions that are based on health needs identified within a community. Each Ophelia project seeks to improve health and equity by increasing the availability and accessibility of health information and services in locally-appropriate ways [41].

### Strength and limitations

The presented study is the first to use HLQ in association with oral health. Another strength of our study might be considered the relatively high response rate (72%).

As a limitation, we consider limited number of oral health indicators, which could be broader and more specific. On the other hand, the general nature of our indicators allows respondents to respond to them without the emergence of recall bias and challenging their abilities related to assessment of the dental procedures. We could also use objective data from dentists, but the higher involvement of dentists might result in higher rate of refusals to participate in our study. Using objective data from the dentist would require different patient consent because it would require agreement with sharing information covered by Personal data protection act. Patients perceive information covered by this Act as very intimate and they are not very willing to disclose it. This is burden, which might result in much lower response rate. The second limitation might be the questionnaire administration in waiting rooms before or after the dentist visit, which may bias the sample towards people who are participating in at least one preventive healthcare activity (dental check-ups) and may, therefore, not include people who are disconnected from health services. The third limitation could be the “self-reported” nature of the oral health data and not using standardized oral health measures or dental health indexes. However, our study brought data about patients’ self-perception of their oral health behaviours, which is also important.

## Conclusion

We found that specific domains of health literacy are associated with most of the oral health outcomes measured in our study. We also found that women and university educated respondents show better oral health-promoting behaviour and higher health literacy is associated with better oral health-promoting behaviour. Our findings showed that age is not associated with the most of the oral health promoting behaviours and health literacy. Focusing on patient’s health literacy might be a plausible method to employ in interventions focusing on oral health improvement in the Slovak population. Our study provides evidence for dentists and other health professional that supporting patient’s health literacy enables them to engage in oral health-promoting behaviour and thus improve their oral health. Increase in health literacy leads to adoption of effective disease-prevention methods, successful adherence to a treatment regimen and ultimately improved oral health-promoting behaviour.

## Additional file

Additional file 1 Oral Health and oral health promoting behaviours items. A list of items used for self-reported oral health indicators and oral health promoting behaviours. (DOCX 14 kb)

## Abbreviations

CI: Confidence interval
HL: Health literacy
HLQ: Health literacy questionnaire
OHPB: Oral health-promoting behaviour
OR: Odds ratio
SD: Standard deviation
SES: Socioeconomic status
