# Supplementary material for: Associations of multidimensional health literacy with reported oral health promoting behaviour among Slovak adults: a cross-sectional study
Source: BMC Oral Health. 2018 Mar 14;18:44. doi: 10.1186/s12903-018-0506-6 (PMC5853055; doi:10.1186/s12903-018-0506-6)
Supplement: Supplementary file 1 — Oral Health and oral health promoting behaviours items. A list of items used for self-reported oral health indicators and oral health promoting behaviours. (DOCX 14 kb) [file 12903_2018_506_MOESM1_ESM.docx]

**Oral Health and oral health promoting behaviours items**

| 1. **What is the most frequent reason for visiting the dentist?** |
| --- |
| 🞎 tooth - ache |
| 🞎 preventive check – up |
| 🞎 making crown, bridge or prosthesis |
| 🞎 to fill the cavities |
| 🞎 endodental treatment (ectraction of the nerve) |
| 🞎 tooth extraction |
| 🞎 dental hygiene |
| 🞎 others: ............................... |

| 1. **Do you use toothpaste with fluoride?** |
| --- |
| 🞎 yes |
| 🞎 no |
| 🞎 I do not know |
| 🞎 I avoid flouride toothpaste |

| 1. **How often do you brush your teeth?** |
| --- |
| 🞎 after each meal |
| 🞎 once a day (morning, afternoon, evening) |
| 🞎 twice a day (morning, evening) |
| 🞎 rarely |

| 1. **Are your gums bleeding when brushing your teeth?** |
| --- |
| 🞎 always |
| 🞎 often |
| 🞎 sometimes |
| 🞎 rarely |
| 🞎 never |

| 1. **Do you use other oral hygiene aids, except toothbrush and toothpaste, do you use?** |
| --- |
| 🞎 electric tooth brush |
| 🞎 interdental tooth brush |
| 🞎 mouth wash |
| 🞎 single brush |
| 🞎 dental floss |
| 🞎 tongue scraper |
